# Trait anxiety impairs cognitive flexibility when overcoming a task acquired response and a preexisting bias

**DOI:** 10.1371/journal.pone.0204694

**Published:** 2018-09-27

**Authors:** Cristina G. Wilson, Amy T. Nusbaum, Paul Whitney, John M. Hinson

**Affiliations:** Department of Psychology, Washington State University, Pullman, WA, United States of America; Technion Israel Institute of Technology, ISRAEL

## Abstract

Individuals with high trait anxiety tend to be worse at flexibly adapting goal-directed behavior to meet changing demands relative to those with low trait anxiety. Past research on anxiety and cognitive flexibility has used tasks that involve overcoming a recently acquired rule, strategy, or response pattern after an abrupt change in task requirements (e.g., choice X led to positive outcomes but now leads to negative outcomes). An important limitation of this research is that many decision making situations require overcoming a preexisting bias (e.g., deciding whether to withdraw a historically winning investment that has experienced recent losses). In the present study we examined whether anxiety differences in the ability to overcome an acquired response extend to the ability to overcome a preexisting bias, when the bias produces objectively disadvantageous decisions. High anxiety (*n* = 78) and low anxiety participants (*n* = 76) completed a commonly used measure of cognitive flexibility, reversal learning, and a novel Framed Gambling Task that assessed the extent to which they could make advantageous decisions when the normatively correct choice was inconsistent with a preexisting framing bias. High anxiety participants showed the expected diminished reversal learning performance and also had poorer ability to make advantageous choices that were inconsistent with the framing bias. Worse performance in the Framed Gambling Task was not driven by poor knowledge of risk contingencies, because high anxiety participants reported the same explicit knowledge as low anxiety participants. Instead, the results suggest high anxiety is associated with general deficits in resolving interference from prepotent responses.

## Introduction

A hallmark of human cognition is its flexibility, i.e., the ability to redirect goal behavior to meet changing demands [1]. Traditionally, cognitive flexibility is measured using task-switching, set-shifting, and reversal learning tasks. In all of these tasks, participants learn an initial response pattern, rule, or strategy that must then be adapted when the contingencies or task requirements are abruptly changed. Typically, contingency/requirement changes are not cued, so participants must learn that a change has occurred through feedback on obtained outcomes. High anxiety individuals display less flexible performance on these tasks by continuing to rely on the acquired response even after learning it is no longer relevant [2–6]. Poor performance in high anxiety individuals is frequently attributed to differences in attentional control, particularly greater interference from the irrelevant response [7].

The relative cognitive inflexibility demonstrated by people with high anxiety can have important consequences for behavior when faced with rapidly changing environments. There are many situations in everyday life where learned information must be inhibited because it is no longer relevant in the decision environment: e.g., preferred commuting routes can be blocked by new construction, and food items in the grocery store can be relocated to different shelves/aisles. There are also situations that require overcoming a preexisting rule-of-thumb or preference that biases decision making. For example, people tend to be risk averse even in situations when taking a risk could result in a better outcome: paying a costly insurance premium for a low probability event or opting out of an experimental health procedure with a strong success rate. An important limitation of existing cognitive flexibility research is that it has almost entirely focused on flexibility when overcoming recently acquired/learned information. However, the ability to flexibly overcome a preexisting bias (like risk-aversion) may be particularly important for high anxiety individuals because research suggests they are more vulnerable to biases than low anxiety individuals [8–9].

It is important to note that people are not risk avoidant in all contexts. Rather there is a strong tendency to be risk averse when choices are framed in terms of gains and to be risk seeking when choices are framed in terms of losses. This framing bias was documented in the classic Asian Disease Problem [10]: when disease outbreak intervention programs were framed in terms of lives saved (i.e., 200 people will be saved OR 1/3 probability 600 people will be saved, 2/3 probability 0 people will be saved) participants preferred the sure option over the gamble option, but when the same programs were framed in terms of lives lost participants preferred the gamble option over the sure option. In business, health, and social domains it is well documented that choice framing can produce risk preference reversals that detrimentally influence decisions [11], and high trait anxiety individuals have been shown to be more vulnerable to framing bias than low anxiety individuals [12–13]. While there is substantial research on the impact of such preexisting biases on decision making, only one study has examined whether framing bias can be flexibly overcome using feedback on obtained outcomes [14], and no research has assessed whether trait anxiety can influence the reduction of bias.

Previous research [14] used a task that combined a framing manipulation with a gambling task to evaluate whether bias could be overcome by learning through outcome feedback. In this task a sure option of either $50 or -$50 was presented along with an ambiguous gamble option. One gamble option had average outcomes greater than the sure gain of $50, and the other gamble option had average outcomes worse than the sure loss of $50. Thus, unlike typical risky choice framing tasks that produce shifts in gamble preference, each choice trial had a normatively correct choice that was sometimes inconsistent with framing bias. For example, when given a choice between the sure gain and the “good” gamble option, framing bias drives selection of the sure gain, but it is more advantageous on average to select the gamble option. It was found that participants developed accurate knowledge of the gains and losses provided by the gamble options but continued to make frame-biased choices, even when the bias led to a normatively incorrect choice [14]. It is reasonable to expect that high trait anxiety will exacerbate the problems with overcoming bias experienced by participants in [14]. High anxiety could increase initial vulnerability to framing bias (as in past research, [12–13]), and anxiety-related changes in attentional control could make it more difficult for high anxiety individuals to adapt to change and overcome their preexisting bias.

**Fig 1:**
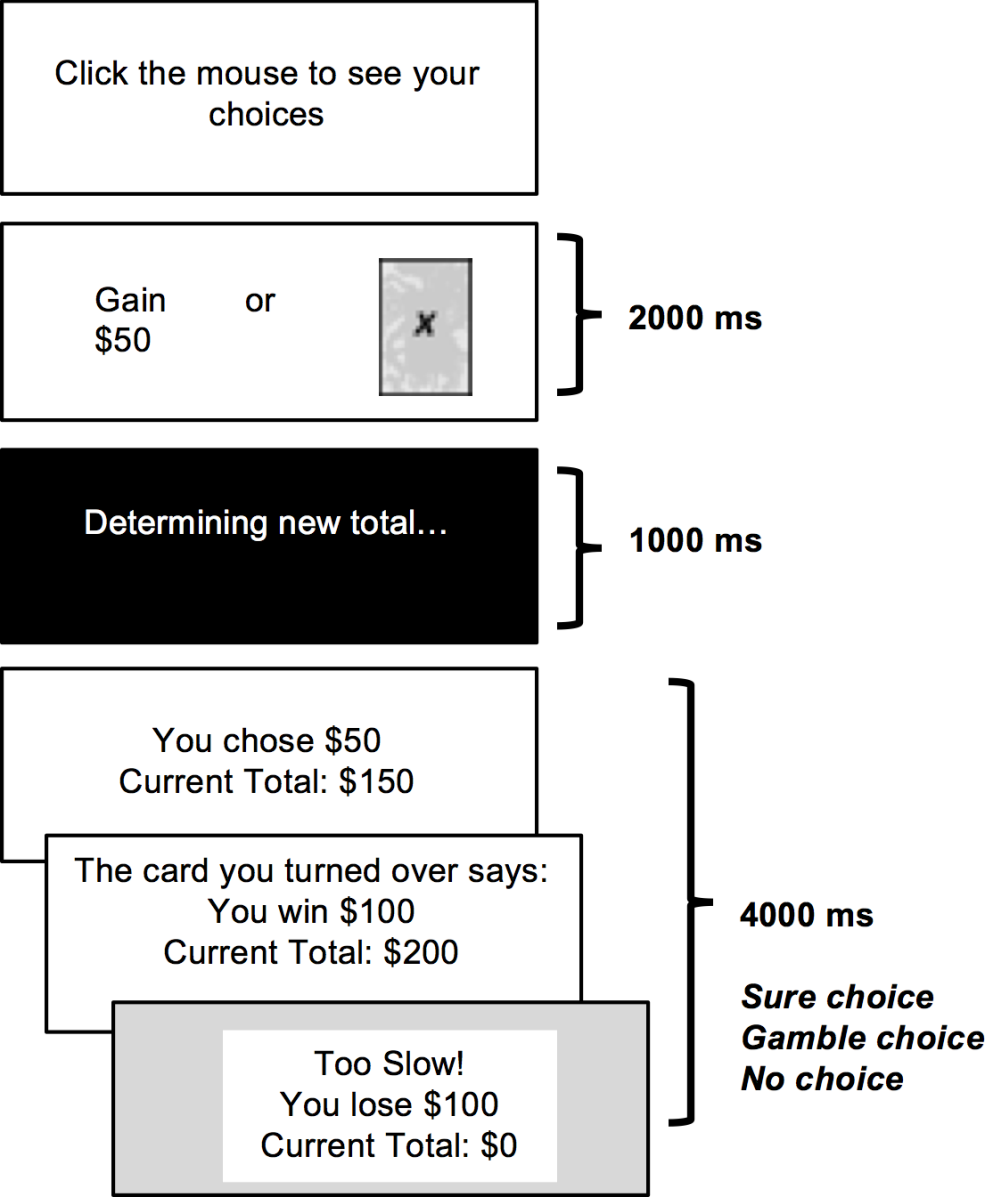
Schematic of Framed Gambling Task choice trial. Each trial consists of a choice between a sure option and a gamble option. If participants make their choice within the given time frame, feedback on their choice (sure or gamble) is provided along with an updated total of their hypothetical monetary winnings. If no choice is made within the time frame then participants are penalized. Timing of trial events is indicated in the figure.

The purpose of the present study was to determine whether the anxiety effects observed with traditional measures of cognitive flexibility extend to the ability to overcome a preexisting bias (in this case a framing bias) when the bias produces objectively disadvantageous decisions. To test this hypothesis, we evaluated anxiety differences in performance on (1) a classic measure of cognitive flexibility, reversal learning, and (2) the previously described Framed Gambling Task (FGT), developed by [14]. In the FGT a sure option (gain or loss) is pitted against one of two gamble options (similar to decks of cards; see Fig 1). Framing bias leads participants to select the sure gain over the gamble option and the gamble option over the sure loss. However, this can lead to normatively incorrect choices because one gamble option (the good deck) is better on average (*M* = + $75) than the sure choice (+/- $50), and the other (the bad deck) is worse on average (*M* = —$75) than the sure choice (Table 1). To maximize advantageous choices participants must learn gamble option contingencies through feedback and then use this knowledge to overcome framing bias. Cognitive flexibility is operationalized as making an increased number of advantageous choices that are inconsistent with the bias, i.e., selecting the good deck over the sure gain, and the sure loss over the bad deck.

**Table 1:** Possible choice trials on the Framed Gambling Task.

#	Sure option	Gamble option	Advantageous choice	Frame-driven choice	Choice requires cognitive flexibility?
1	Gain $50	Deck X (Bad)	Gain $50	Gain $50	No
2	Gain $50	Deck Y (Good)	Deck Y (Good)	Gain $50	Yes
3	Lose $50	Deck X (Bad)	Lose $50	Deck X (Bad)	Yes
4	Lose $50	Deck Y (Good)	Deck Y (Good)	Deck Y (Good)	No

Each deck contains gains and losses based on a fixed set of nine independent outcomes. Deck Y, the good deck, contains outcomes sampled from a normal distribution with a mean of +$75 and a standard deviation of 100 (-100, -55, -30, 40, 115, 140, 160, 180, 195). The average gain from the good deck is approximately $138, and the average loss is approximately $61. Deck X, the bad deck, contains outcomes sampled from a normal distribution with a mean of -$75 and a standard deviation of 100 (-200, -180, -135, -125, -110, -80, -60, 85, 95). The average loss from the bad deck is approximately $127, and the average gain is approximately $90. The advantageous (normatively correct) choice on any trial is to choose the good deck or avoid the bad deck. Cognitive flexibility is required on trials where the advantageous choice is inconsistent with framing bias.

We expected to replicate the established finding of poorer cognitive flexibility amongst high anxiety participants, using reversal learning as a standard measure of cognitive flexibility. Our primary interest, however, was in whether similar effects of anxiety would be obtained when flexibility involved overcoming the preexisting framing bias in the FGT. We hypothesized that high anxiety participants would initially be more vulnerable to framing bias in the FGT and demonstrate a reduced ability to overcome bias, i.e., fewer bias-inconsistent advantageous choices over time. If high anxiety participants have more difficulty making bias-inconsistent choices then there are two general possibilities for why this effect could be obtained. In the FGT, determining whether the deck option is better or worse than the sure option involves accumulating probabilistic outcome feedback over a series of trials. High anxiety could interfere with the ability to accumulate outcome feedback, which is necessary to determine whether the deck option is better or worse than the sure option. If this occurs, then anxiety differences in choice behavior would be due to high anxiety participants having poorer knowledge of the expected outcomes for each deck. Alternatively, high anxiety could impair the ability to use attentional control to prevent or overcome interference from the bias-driven response (see [15–16] for a parallel claim about the source of anxiety effects in traditional cognitive flexibility measures). If this occurs, then anxiety differences would be present in choice behavior even when knowledge of deck outcomes is equivalent between high and low anxiety participants. To evaluate the possible sources of anxiety differences in the FGT we included knowledge probes in the task asking participants to rate the valence and estimate the average gain and loss from each deck.

## Methods

### Participants

There were 237 adult participants drawn from an undergraduate student population (60% female, 40% male). Students had to be at least 18 years of age to participate (*M* = 20.18, *SD* = 2.56). Participants were recruited via the Department of Psychology Human Subject Pool, a web-based experiment sign-up system available to students registered in psychology courses. Participants were awarded credits through the Human Subject Pool as compensation for their participation. Credits were redeemable for points in psychology courses.

### Procedure & materials

All procedures and materials were approved by the Washington State University Institutional Review Board, including the informed consent document which was provided to each participant for signature by a trained research assistant. At the end of the experimental session, participants were debriefed by the same research assistant. During the session participants were seated at a computer work station to complete an hour and a half task battery consisting of a Go/No-Go (GNG) reversal learning task, the FGT, and a series of questionnaires on anxiety. The order of tasks was random, but the questionnaires were always completed last to minimize demand characteristics. The questionnaires were administered through Qualtrics (Qualtics, Provo, UT). In addition to questionnaires on anxiety, questionnaires related to substance use and eating behavior were included as part of a separate project. The State-Trait Anxiety Inventory (STAI) was used to establish participants’ level of trait anxiety [17]. Participants who scored below 38 were categorized as low anxiety (*n* = 76) and those who scored above 45 were categorized as high anxiety (*n* = 78; Table 2). Cutoffs were determined by computing tertiles of trait anxiety scores, and were similar to cutoffs used in previous research [2–3]. Sample sizes were sufficient to have 80% power to detect the contrasts of interest between the high and low anxiety groups, assuming small effect sizes, i.e., Cohen’s d of .20 to .30 [18].

**Table 2:** Characteristics of low anxiety and high anxiety samples.

	Low Anxiety		High Anxiety	
	*M (SD)*	Freq.	*M (SD)*	Freq.
N		76		78
Sex(male/female)		41 / 35		22 / 56
Age	20.16 (2.29)		20.57 (3.39)	
STAI-T	31.32 (4.38)		53.67 (6.66)	
STAI-S	31.11 (7.34)		45.74 (9.65)	

The ratio of males to females in our high and low anxiety samples was skewed such that the high anxiety sample was predominately female and the low anxiety sample was predominantly male. This is consistent with a wealth of evidence that excessive anxiety is approximately twice as prevalent in females as males [19–21]. To rule out a possible interaction of sex and anxiety, all analyses were run with both variables included as between-subjects factors. No significant interactions of sex and anxiety were found.

#### Go/No-Go reversal learning task

The GNG reversal learning task was programmed using E-Prime 2.0 software (Psychology Software Tools, Pittsburgh, PA). In the task, a two-digit number is presented (e.g., 16) and participants must learn through trial and error whether that number is associated with a “go” response or a “no-go”, i.e., withheld response. There are 8 possible numbers, 4 go stimuli (16, 11, 97, 78) and 4 no-go stimuli (86, 17, 83, 42). Over half-way through the task, without warning, contingencies change and the numbers that were previously associated with a go response become associated with a no-go response, and vice versa. A go response was executed by pressing the space bar on a standard keyboard. Participants had 750 milliseconds to make their choice. Participants then received feedback about their response indicating whether they were correct or incorrect.

There were 104 total trials. The order presentation of digit stimuli was random, but each stimulus was presented once every 8 trials. The first 64 trials of the task were the learning phase, with participants experiencing each stimulus 8 times. The reversal occurred on trial 65 and the subsequent 7 trials were the reversal phase, with participants experiencing each stimulus once. The final 32 trials were the recovery phase, with participants experiencing each stimulus 4 times. Cognitive flexibility was operationalized as the ability to improve discrimination of go/no-go stimuli during the recovery phase.

#### Framed Gambling Task

The FGT was programmed with E-Prime 2.0 software (Psychology Software Tools, Pittsburgh, PA). The task consists of 72 trials in which participants choose between a gamble and a sure option. The gamble option is either “Deck X” or “Deck Y”, while the sure option is either a gain or a loss of $50, creating four different types of choices (Table 1). Advantageous performance requires learning through feedback that one deck is good, leading most often to gains, and one deck is bad, leading most often to losses. Importantly, the average outcome from the good deck is $75 and the average outcome from the bad deck is -$75. Thus, on any given trial there is a normatively correct option, given that the good deck is better than the sure gain and the bad deck is worse than the sure loss.

Each deck was a different color and labeled either “X” or “Y.” Deck options were pitted against a sure gain and a sure loss equally often. Once the choice options appeared, participants had two seconds to select their choice using a computer mouse, with a left click selecting the sure option and a right click selecting the gamble. If a participant made a choice within the two seconds they received feedback for their selection as well as an updated total of their winnings. If no response was detected within the two seconds, participants were informed they were too slow and were penalized $100: this occurred on approximately 1% of trials in both anxiety groups.

To assess participants’ knowledge of deck outcomes, we asked them to rate each deck on a scale from -10 (Terrible) to +10 (Excellent), and estimate the average gain and loss from each deck. Estimations and ratings were collected every 18 trials, a total of 4 times.

## Results

First we examined performance on the GNG reversal learning task to determine whether our high anxiety sample had the predicted problems with overcoming an acquired response pattern. Signal detection theory was used to analyze the GNG task because it provides an efficient way of summarizing how decisions are made when there is some amount of uncertainty (in this case uncertainty about which stimuli are associated with “go” versus “no-go” responses). Hits and false alarms were used to compute the signal detection parameter of sensitivity (*d'*), where a hit is making a “go” response on a “go” trial and a false alarm is making a “go” response on a “no-go” trial. A larger *d'* reflects a greater ability to discriminate go stimuli from no-go stimuli in the task, i.e., high hits, low false alarms.

**Fig 2:**
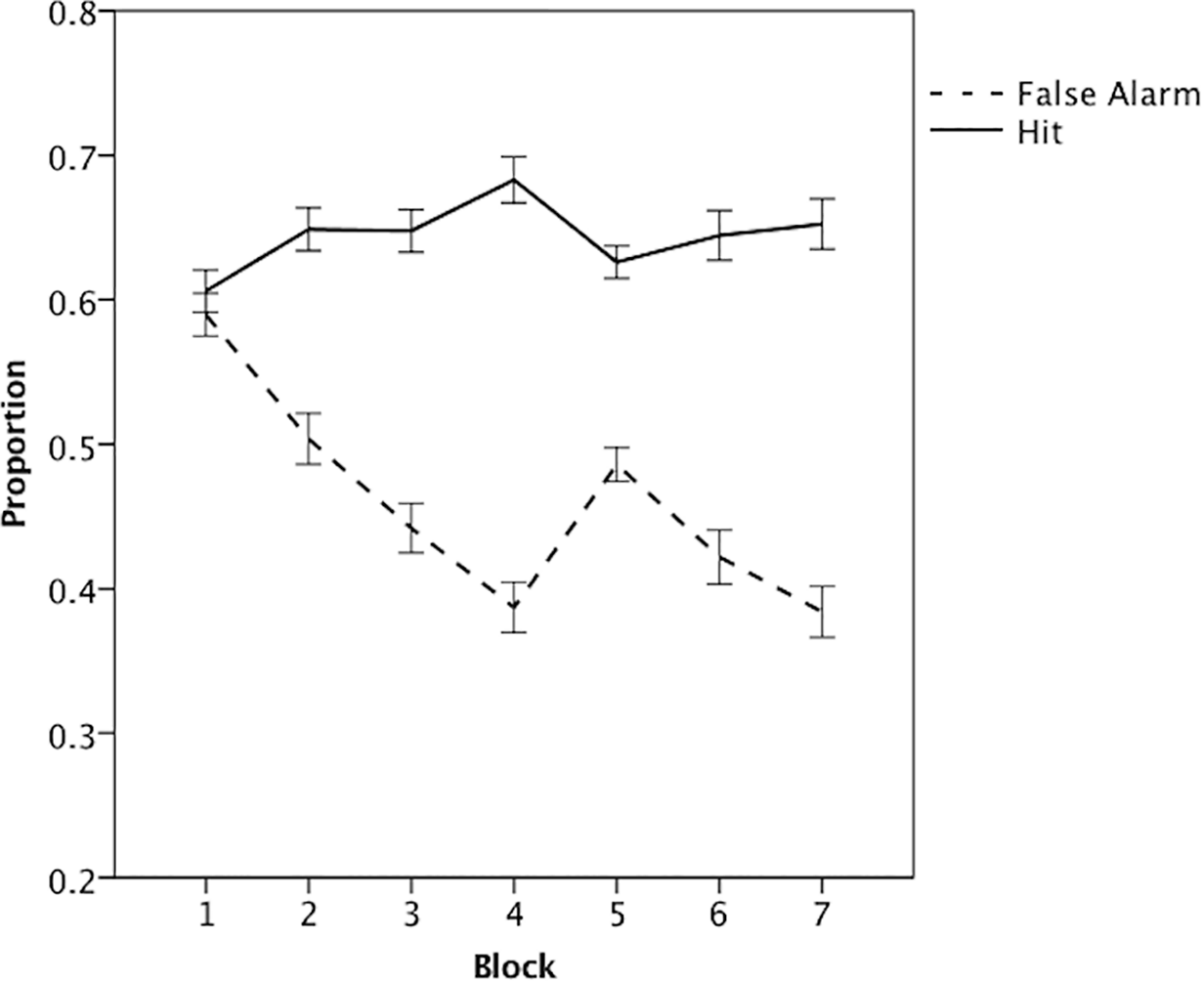
The proportion of hits and false alarms on the Go/No-Go reversal learning task collapsed across anxiety groups. The proportion of hits (solid line) and false alarms (dotted line) across the learning phase (block 1–4), reversal phase (block 5), and recovery phase (block 6–7). Error bars are +/- 1 standard error.

**Fig 3:**
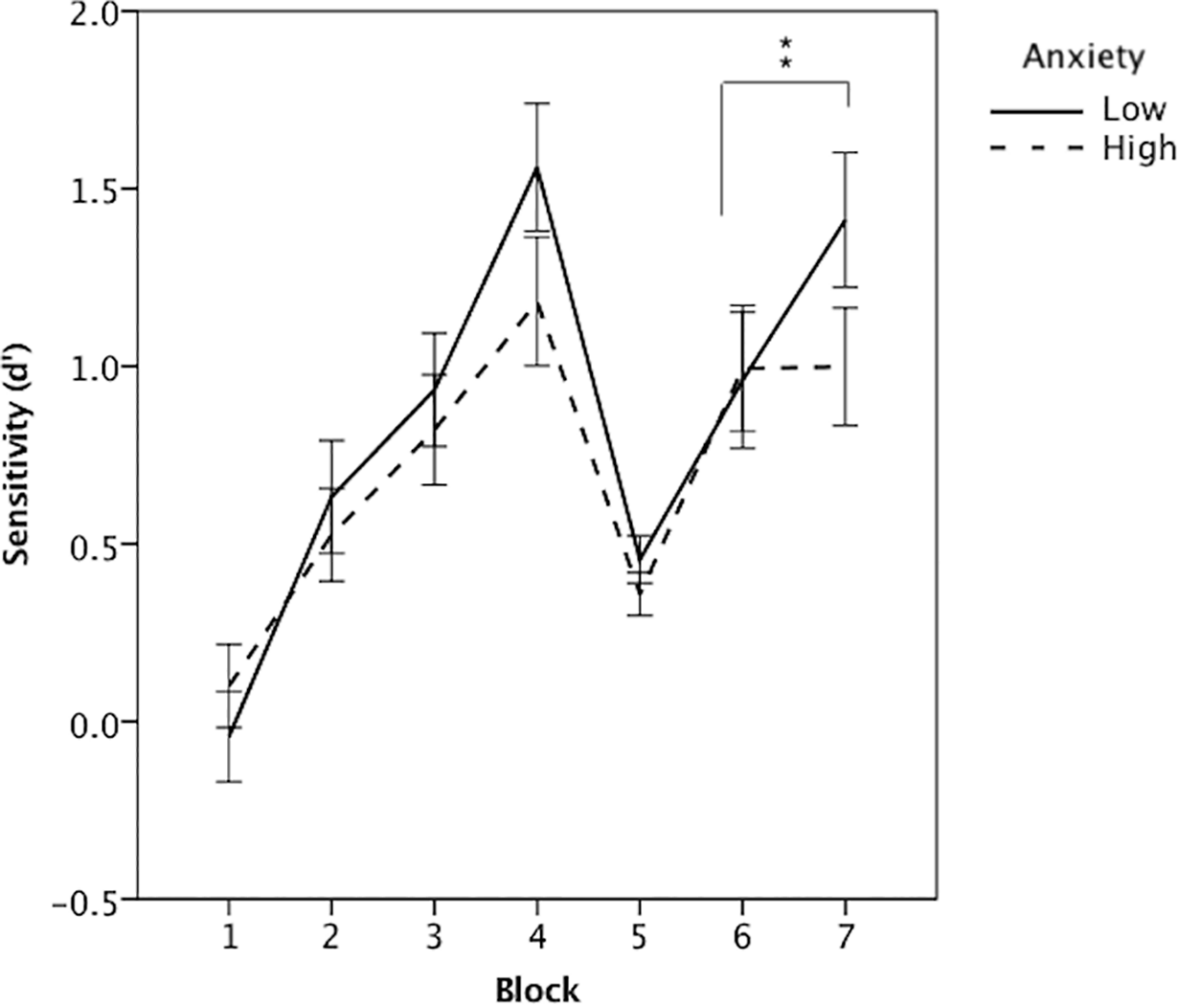
Sensitivity (*d’*) on the Go/No-Go reversal task. Sensitivity across the learning phase (block 1–4), reversal phase (block 5), and recovery phase (block 6–7) between low anxiety (solid line) and high anxiety participants (dotted line). Error bars are +/- 1 standard error.

Overall, our participants showed the expected pattern of performance. In the learning phase, hits steadily increased and false alarms decreased resulting in improved *d'*. At reversal, hits and false alarms converged moderately, with hits decreasing and false alarms increasing resulting in a drop in *d'*., Finally, in the recovery phase, as participants adapted to the change, hits again increased and false alarms decreased resulting in improved *d'* (Figs 2 and 3). Cognitive flexibility was assessed by analyzing improvements in *d'* over the recovery phase: *d'* in the first half of the recovery phase was subtracted from *d'* in last half. As predicted, an independent samples t-test showed that the high anxiety group had a reduced ability to flexibly overcome the acquired response pattern, *t* (152) = 1.99, *p* = .049, *d* = 0.32. High anxiety participants (*M* = 0.004, *SD* = 1.22) demonstrated less improvement in the recovery phase than the low anxiety group (*M* = 0.45, *SD* = 1.56).

**Fig 4:**
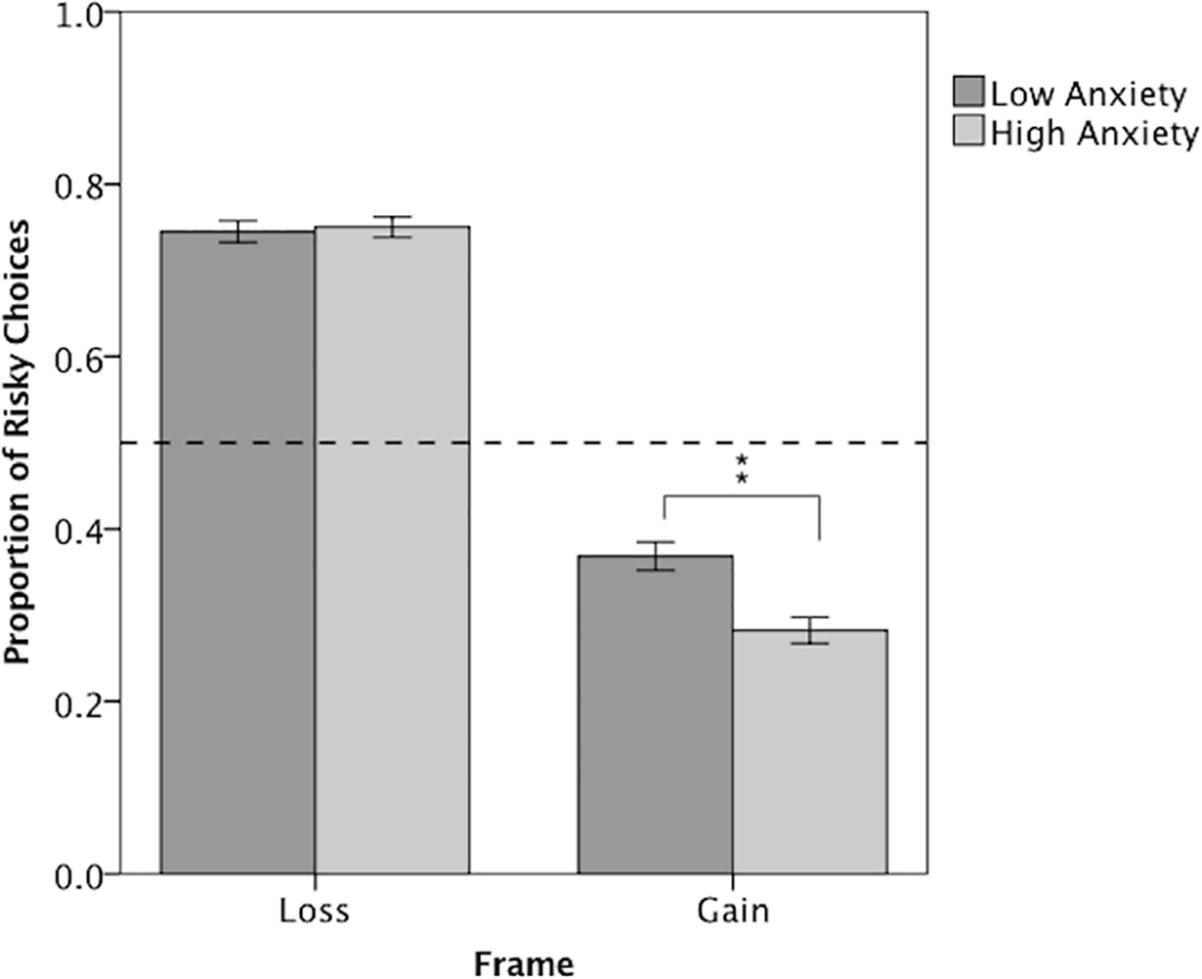
The proportion of gambles on the Framed Gambling Task. The proportion of gambles made in the loss frame (left panel) and gain frame (right panel), between low anxiety (dark gray) and high anxiety participants (light gray). Error bars are +/- 1 standard error. A reference line at .50 indicates indifference between frames. Deviations below .5 in the Gain Frame and above .5 in the Loss Frame indicate the magnitude of bias.

Next we examined performance on the FGT to determine whether trait anxiety influenced the ability to flexibly overcome bias. To ensure the FGT created the expected framing bias, we assessed the proportion of gamble choices when faced with a sure loss versus a sure gain. A 2 (Frame: Gain, Loss) x 2 (Anxiety: High, Low) repeated measures ANOVA revealed participants chose the gamble options more frequently when faced with the loss frame versus the gain frame, exhibiting the expected bias, *F* (1, 152) = 403.26, *MSE* = 0.03, *p* < 0.01, *η*_*p*_^*2*^ = 0.73 (Fig 4). A Frame X Anxiety interaction, *F* (1, 152) = 4.71, *p* = 0.03, *η*_*p*_^*2*^ = 0.03, broken down using an independent samples t-test showed the high anxiety group was more risk-averse in the gain frame than the low anxiety group, *t* (152) = 2.62, *p* = 0.01, *d* = 0.43. Despite this, anxiety groups did not differ in their initial proportion of advantageous choices. A 2 (Bias: Consistent, Inconsistent) X 2 (Anxiety) repeated measures ANOVA of advantageous choices in the first 24 trials showed all participants initially made a higher proportion of advantageous choices that were consistent with bias versus inconsistent, *F* (1, 152) = 378.98, *MSE* = 0.05, *p* < 0.01, *η*_*p*_^*2*^ = 0.71. Therefore, although the high anxiety group was slightly more vulnerable to risk-aversion in the gain frame, both anxiety groups showed the same initial pattern of bias-driven advantageous choices.

**Fig 5:**
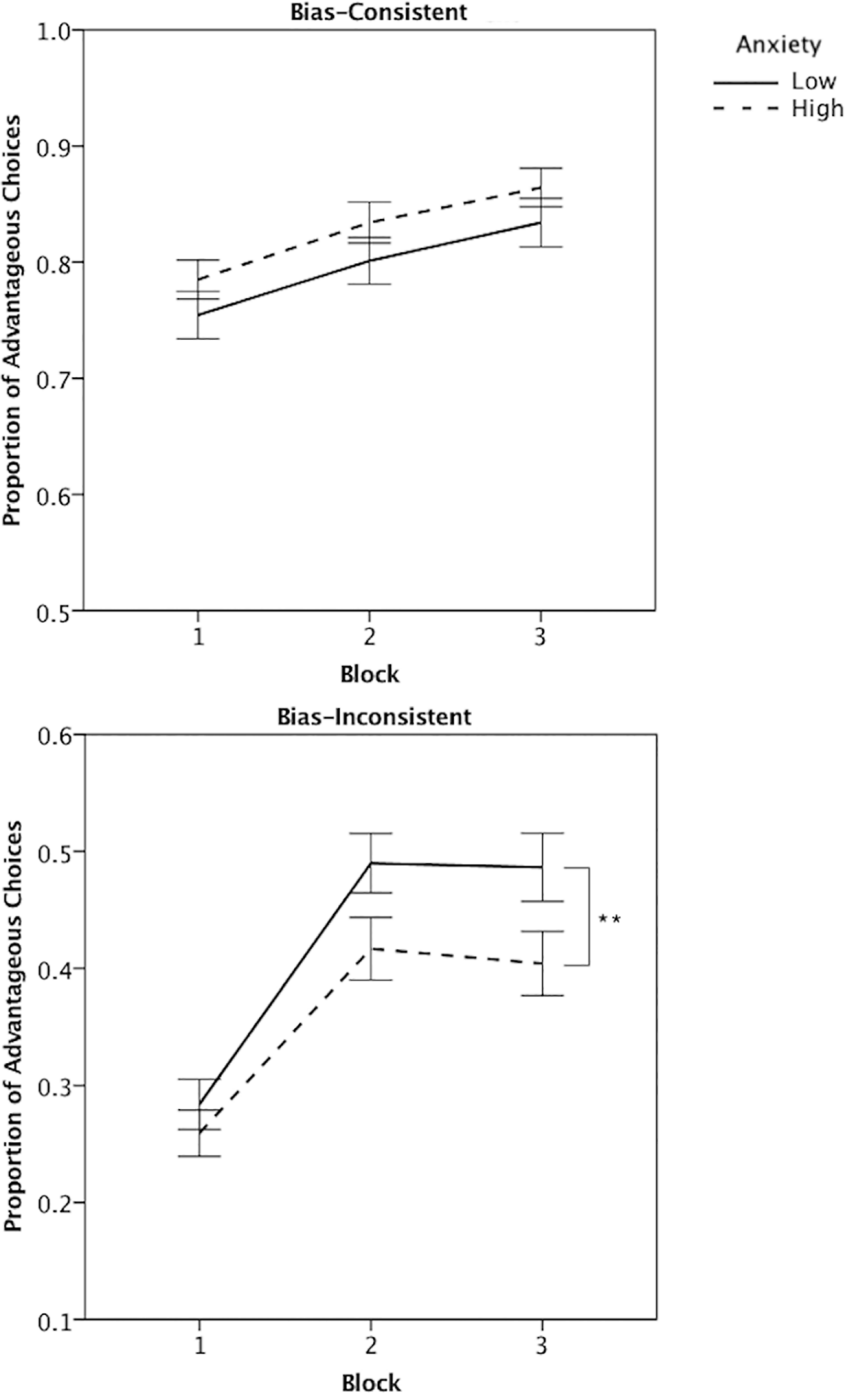
The proportion of advantageous choices on the Framed Gambling Task. The proportion of advantageous choices made that were consistent with bias (top) and inconsistent with bias (bottom), across 3 blocks of 24-trials, between low anxiety (solid line) and high anxiety participants (dotted line). Error bars are +/- 1 standard error.

To determine whether the anxiety groups differed in their ability to overcome bias we analyzed the proportion of advantageous choices that were consistent versus inconsistent with the frame-like bias across trial blocks. A 2 (Bias) X 3 (Trial block: 1–24, 25–48, 49–72) X 2 (Anxiety) repeated measures ANOVA of advantageous choices found a significant interaction of Bias X Block, *F* (2, 304) = 13.93, *MSE* = 0.03, *p* < 0.01, *η*_*p*_^*2*^ = 0.08. This was broken down by repeated measures ANOVAs of Block, which showed that both anxiety groups improved advantageous choices that were inconsistent with bias, *F* (2, 304) = 43.16, *MSE* = 0.04, *p* < 0.01, *η*_*p*_^*2*^ = 0.22, to a greater extent than bias-consistent choices, *F* (2, 304) = 16.24, *MSE* = 0.02, *p* < 0.01, *η*_*p*_^*2*^ = 0.10 (Fig 5). A significant Bias X Anxiety interaction, *F* (1, 152) = 4.71, *MSE* = 0.10, *p* = 0.03, *η*_*p*_^*2*^ = 0.03, broken down using an independent samples t-test showed high and low anxiety participants made a similar proportion of bias-consistent advantageous choices, *t* (152) = -1.41, *p* = 0.16, *d* = 0.23, but the high anxiety group made fewer bias-inconsistent advantageous choices, *t* (152) = 1.89, *p* = 0.05, *d* = 0.31. Thus, while both groups improved choices over time, the low anxiety group overcame bias to a greater degree than the high anxiety group.

To evaluate whether anxiety differences in the FGT were the result of differences in knowledge of gamble option contingencies, we next analyzed participants’ responses to knowledge probe questions. Preliminary analyses using a repeated measures ANOVA showed there was no interaction of Anxiety X Block on probe responses. Both anxiety groups improved the accuracy of their estimates and ratings over time. As a result, probe responses were averaged across blocks for analysis. A multivariate ANOVA (MANOVA) found no anxiety differences in average estimates or ratings of the deck options (Table 3). Both anxiety groups had moderately accurate estimates of gain/loss outcomes and valence ratings that discriminated between the good and bad deck.

**Table 3:** Ratings and estimations of the Framed Gambling Task gamble options.

	Low Anxiety	High Anxiety	MANOVA
	*M (SD)*	*M (SD)*	p-value
*Good Deck*			
Rating	3.75 (2.86)	3.74 (3.32)	= .985
Average Gain	117.18 (83.49)	102.02 (59.42)	= .197
Average Loss	-72.35 (39.10)	-75.12 (81.46)	= .790
*Bad Deck*			
Rating	-2.89 (4.04)	-2.18 (4.99)	= .331
Average Gain	42.87 (39.28)	48.40 (51.20)	= .455
Average Loss	-110.13 (64.20)	-97.55 (60.06)	= .231

Ratings were on a scale of -10 (Terrible) to +10 (Excellent). The true (and experienced) average values for the good deck were a gain of 138 (138.72) and a loss of 61 (61.63). The true (and experienced) average values for the bad deck were a loss of 127 (127.29) and a gain of 90 (89.44).

## Discussion

The present study built on existing anxiety and cognitive flexibility research by examining anxiety differences in a novel form of flexibility, the ability to overcome a preexisting bias. The ability to overcome a preexisting bias is important in many of the situations we must adapt to in everyday life, perhaps even more so than flexibility defined in terms of overcoming a recently acquired response tendency. Since high anxiety individuals have poor performance on traditional measures of cognitive flexibility [2–6], it was hypothesized that they would also be less able to overcome a preexisting framing bias. Consistent with this hypothesis, our high anxiety sample demonstrated impaired performance on a traditional flexibility task (reversal learning), and were worse at reducing framing bias on the FGT. High anxiety participants had poorer flexibility in the FGT despite reporting knowledge of gamble option contingencies that was comparable to low anxiety participants.

The predominant theory of anxiety differences in traditional measures of cognitive flexibility comes from Attentional Control Theory [15–16]. According to Attentional Control Theory, trait anxiety increases activation of bottom-up (stimulus-driven) attention and decreases activation of top-down (goal-driven) attention. Because of this, high anxiety individuals are susceptible to continued interference from salient stimuli that are potentially irrelevant. Thus, from this perspective, an anxiety-related deficit in FGT performance is reflective of a diminished ability to prevent or overcome interference from a bias-related prepotent response. This would explain why the high anxiety group had poorer cognitive flexibility despite having similar knowledge of gamble options as the low anxiety group–they were simply more vulnerable to interference from the prepotent response.

While Attentional Control Theory offers a reasonable explanation of our findings, it is worthwhile to consider other sources of anxiety effects on FGT performance. Cognitive flexibility encompasses multiple processes, e.g., representing a rule/strategy/pattern, monitoring and learning from feedback, preventing or resolving conflict between competing response tendencies, managing the uncertainty of decision outcomes. Because not all processes are the same for all flexibility measures, it is possible (and likely) that the effects of anxiety are task dependent [22]. Here we discuss the potential influence of two information processing biases on FGT performance, both frequently associated with high anxiety: sensitivity to negative outcomes and negative interpretation of uncertainty [8–9].

Sensitivity to negative outcomes and negative interpretation of uncertainty are biases that influence the way high anxiety individuals transform the probabilities of gains/losses into decision values (and weight those values). Sensitivity to negative outcomes makes high anxiety individuals more averse to large losses [23], while the negative interpretation of uncertainty leads high anxiety individuals to favor certain and safe choice options over risky alternatives [24]. Some combination of these biases may help to explain the pattern of results found among our high anxiety group. The tendency of the high anxiety group to avoid the sure loss (even when gambling led to a probabilistically worse outcome), could be driven by a greater sensitivity to negative outcomes. The tendency to choose the sure gain over the deck options (even when gambling led to a probabilistically better outcome), could be driven by the negative interpretation of uncertainty. If this is the case, then anxiety differences in performance on the FGT may not be exclusively the product of attentional control differences, but, rather, the result of information processing differences in the weighting of decision values.

In sum, the results of the present study provide evidence that trait anxiety is associated with a reduced ability to adapt to changing circumstances, both when overcoming an acquired response (GNG reversal learning) and a preexisting bias (FGT). In future research, it will be important to investigate the ability to overcome a preexisting bias in psychiatric conditions. Current research suggests the progression from normal to clinical forms of anxiety is a continuum, with high trait anxiety individuals at increased risk of developing anxiety disorders [25] and substance use disorders [26]. Clinically anxious populations have poor performance on traditional measures of cognitive flexibility, like task-switching [27] and reversal learning [28], but it is unclear whether this deficit extends to the ability to overcome a preexisting bias. We acknowledge that the generalizability of the results beyond a non-clinical sample is a limitation of the current study. However, our findings advance the literature by demonstrating that the influence of trait anxiety on cognitive flexibility (as traditionally defined) extends to the ability to overcome a preexisting bias. Teasing apart the processes that drive anxiety effects in clinical and non-clinical populations when overcoming a preexisting bias, and determining whether they differ from the source of anxiety effects when overcoming an acquired response is an interesting avenue for future research, particularly given the importance of cognitive flexibility in everyday decision making.
